# Supplementary material for: Coronavirus M Protein Trafficking in Epithelial Cells Utilizes a Myosin Vb Splice Variant and Rab10
Source: Cells. 2024 Jan 10;13(2):126. doi: 10.3390/cells13020126 (PMC10814003; doi:10.3390/cells13020126)
Supplement: Supplementary file 1 [file cells-13-00126-s001.zip › cells-2778719-supplementary.pdf]

SUPPLEMENTAL FIGURES

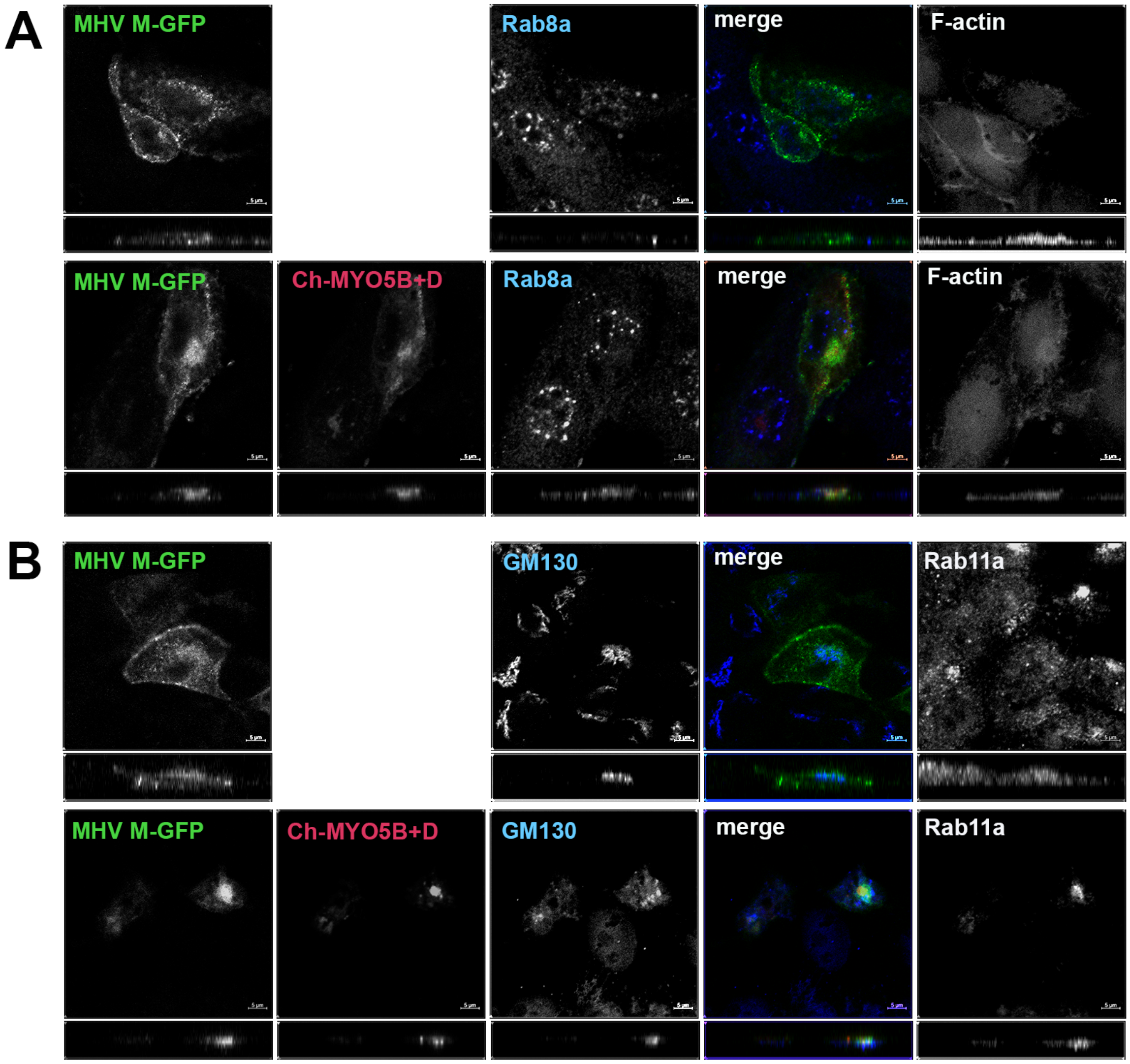

Supplemental Figure S1: Localization of MHV M-GFP with Cherry-MYO5B+D with Rab8a and the Golgi apparatus. A. Expression of MHV M-GFP alone and with Cherry-MYO5B+D in MDCK cells with immunostaining for endogenous Rab8a (cyan) and F-actin (Phalloidin, blue). B. Expression of MHV M-GFP alone and with Cherry-MYO5B+D in MDCK cells with immunostaining for endogenous Rab11a (cyan) and GM130 (blue) as a marker of the Golgi apparatus. Z axis projections are shown below X-Y slice images, with the merged overlap at the right. Bar = 5 µm. Results are representative of 3 individual experiments.

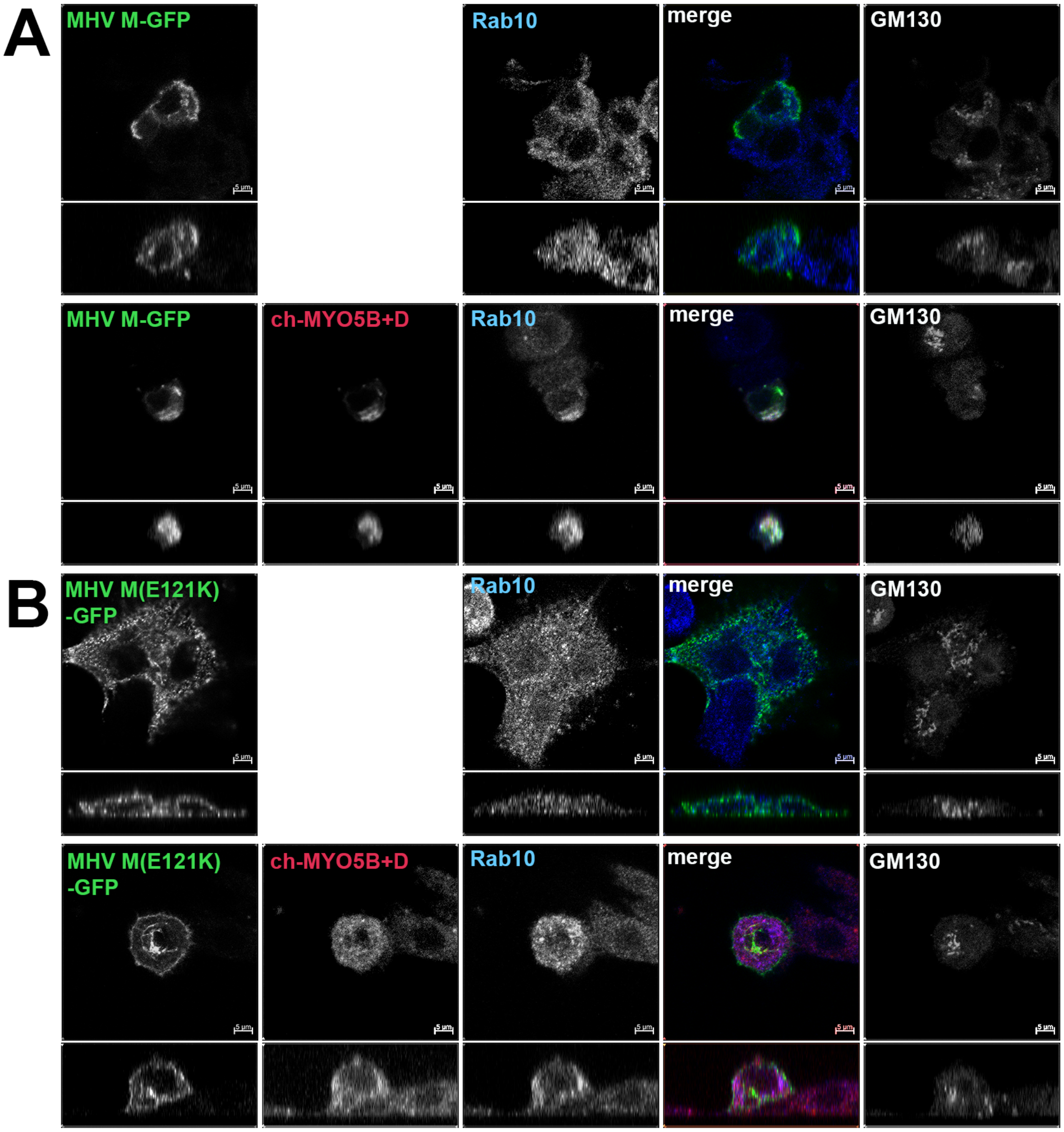

Supplemental Figure S2: E121K point mutant in MHV M cytoplasmic tail blocks co-localization with co-expressed Cherry-MYO5B+D in A549 cells. A549 lung cells were transfected with A) MHV M-GFP or B) MHV M(E121K)-GFP without or with MYO5B+D. All cells were co-immunostained for endogenous Rab10 and GM130 as a marker of the Golgi apparatus. Labels on the individual panels indicate the color used to produce the three-color merged image. Panels to the right of the merged panel were not used in the production of the merged image. Z axis projections are shown below X-Y slice images, with the merged overlap at the right. Bar = 5 μm. Results are representative of 3 individual experiments.

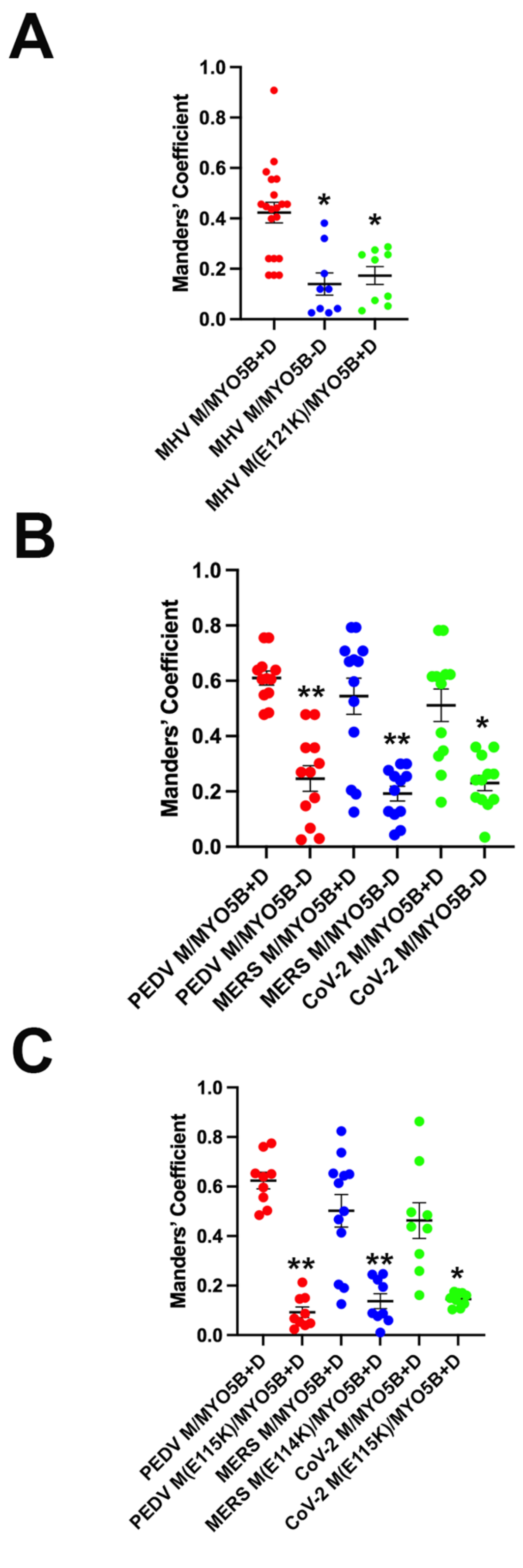
A
Manders' Coefficient
1.0
0.8
0.6
0.4
0.2
0.0
*
*
MHV M/MYO5B+D
MHV M/MYO5B-D
MHV M(E121K)/MYO5B+D
B
Manders' Coefficient
1.0
0.8
0.6
0.4
0.2
0.0
**
**
*
PEDV M/MYO5B+D
PEDV M/MYO5B-D
MERS M/MYO5B+D
MERS M/MYO5B-D
CoV-2 M/MYO5B+D
CoV-2 M/MYO5B-D
C
Manders' Coefficient
1.0
0.8
0.6
0.4
0.2
0.0
**
**
*
PEDV M/MYO5B+D
PEDV M(E115K)/MYO5B+D
MERS M/MYO5B+D
MERS M(E114K)/MYO5B+D
CoV-2 M/MYO5B+D
CoV-2 M(E115K)/MYO5B+D

Supplemental Figure S3: Colocalization analysis for dual expression of M proteins with MYO5B in A549 cells. 3-dimensional Manders' coefficients were calculated for Z-stack images for dually transfected A549 cells. Dual expression pairs are noted on the X-axis. A. MHV M-GFP expression with MYO5B+D or MYO5BΔD (MYO5B-D). N≥9. *p<0.001 vs MHV M expressed with MYO5B+D. **p<0.0001 vs MHV M expressed with MYO5B+D. B. M-GFP proteins from PEDV, MERS and SARS-CoV-2 co-expressed with either MYO5B+D or MYO5BΔD (MYO5B-D). **p<0.0001 vs M protein expressed with MYO5B+D. N≥9. C. Colocalization of Cherry-MYO5B+D with M-GFP chimeras for PEDV, MERS and SARS-CoV-2 compared with E to K mutants of each M protein. N≥6. For A, *p<0.001 versus MHV M co-expressed with MYO5B+D. For B, *p<0.001 between wild type M protein expressed with MYO5B+D vs MYO5B-D. **p<0.0001 between wild type M protein expressed with MYO5B+D vs MYO5B-D. For C, *p<0.001 between mutant and wild type M protein expressed with MYO5B+D. **p<0.0001 between mutant and wild type M protein expressed with MYO5B+D. Analysis was performed on a minimum of 3 fields from at least 3 separate experiments.

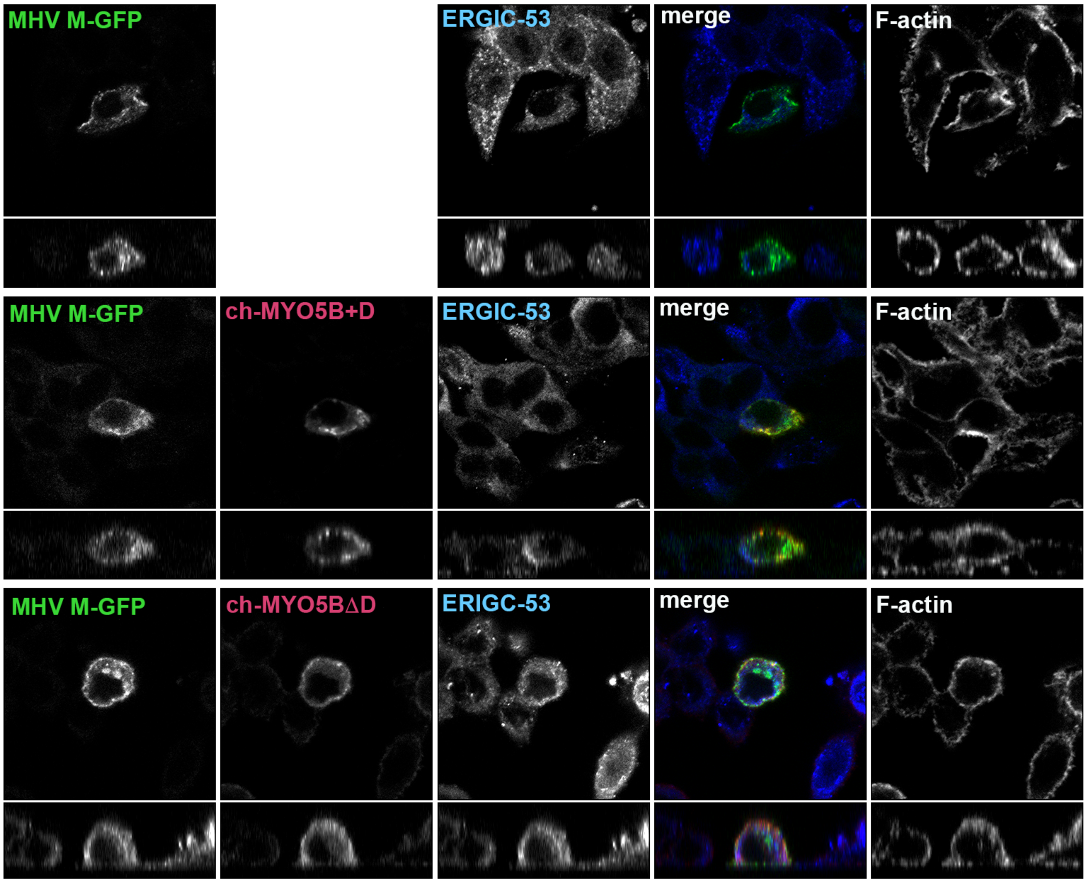

Supplemental Figure S4: Localization of MHV M-GFP with Cherry-MYO5B+D co-expressed in A549 lung cells. MHV M-GFP was expressed in A549 lung cells alone, with Cherry-MYO5B+D or with Cherry-MYO5BΔD. All cells were immunostained for ERGIC-53 and for F-actin with fluorescent phalloidin. Labels on the individual panels indicate the color used to produce the three-color merged image. Panels to the right of the merged panel were not used in the production of the merged image. Z axis projections are shown below X-Y slice images, with the merged overlap at the right. Bar = 5 µm. Results are representative of 3 individual experiments.

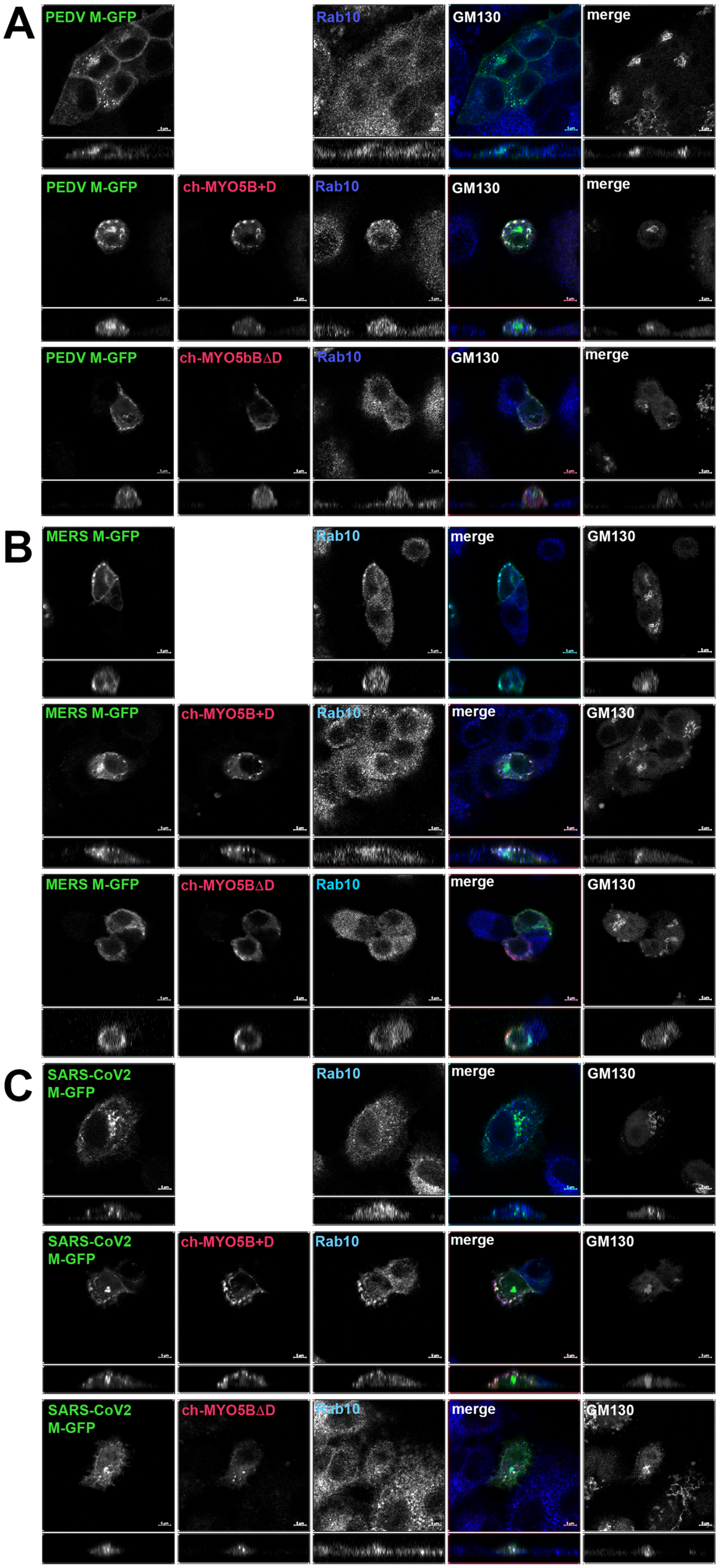

A
PEDV M-GFP
Rab10
GM130
merge
PEDV M-GFP
ch-MYO5B+D
Rab10
GM130
merge
PEDV M-GFP
ch-MYO5bBΔD
Rab10
GM130
merge
B
MERS M-GFP
Rab10
merge
GM130
MERS M-GFP
ch-MYO5B+D
Rab10
merge
GM130
MERS M-GFP
ch-MYO5BΔD
Rab10
merge
GM130
C
SARS-CoV2 M-GFP
Rab10
merge
GM130
SARS-CoV2 M-GFP
ch-MYO5B+D
Rab10
merge
GM130
SARS-CoV2 M-GFP
ch-MYO5BΔD
Rab10
merge
GM130

Supplemental Figure S5: Co-localization of coronavirus M proteins with co-expressed MYO5B+D in A549 cells. A) PEDV M-GFP, B) MERS M-GFP and C) SARS-CoV-2 M-GFP were expressed in A549 lung cells alone, with Cherry MYO5B+D or Cherry-MYO5BΔD. All cells were co-immunostained for endogenous Rab10 and GM130 as a marker of the Golgi apparatus. Labels on the individual panels indicate the color used to produce the three-color merged image. Panels to the right of the merged panel were not used in the production of the merged image. Z axis projections are shown below X-Y slice images, with the merged overlap at the right. Bar = 5 µm. Results are representative of 3 individual experiments.

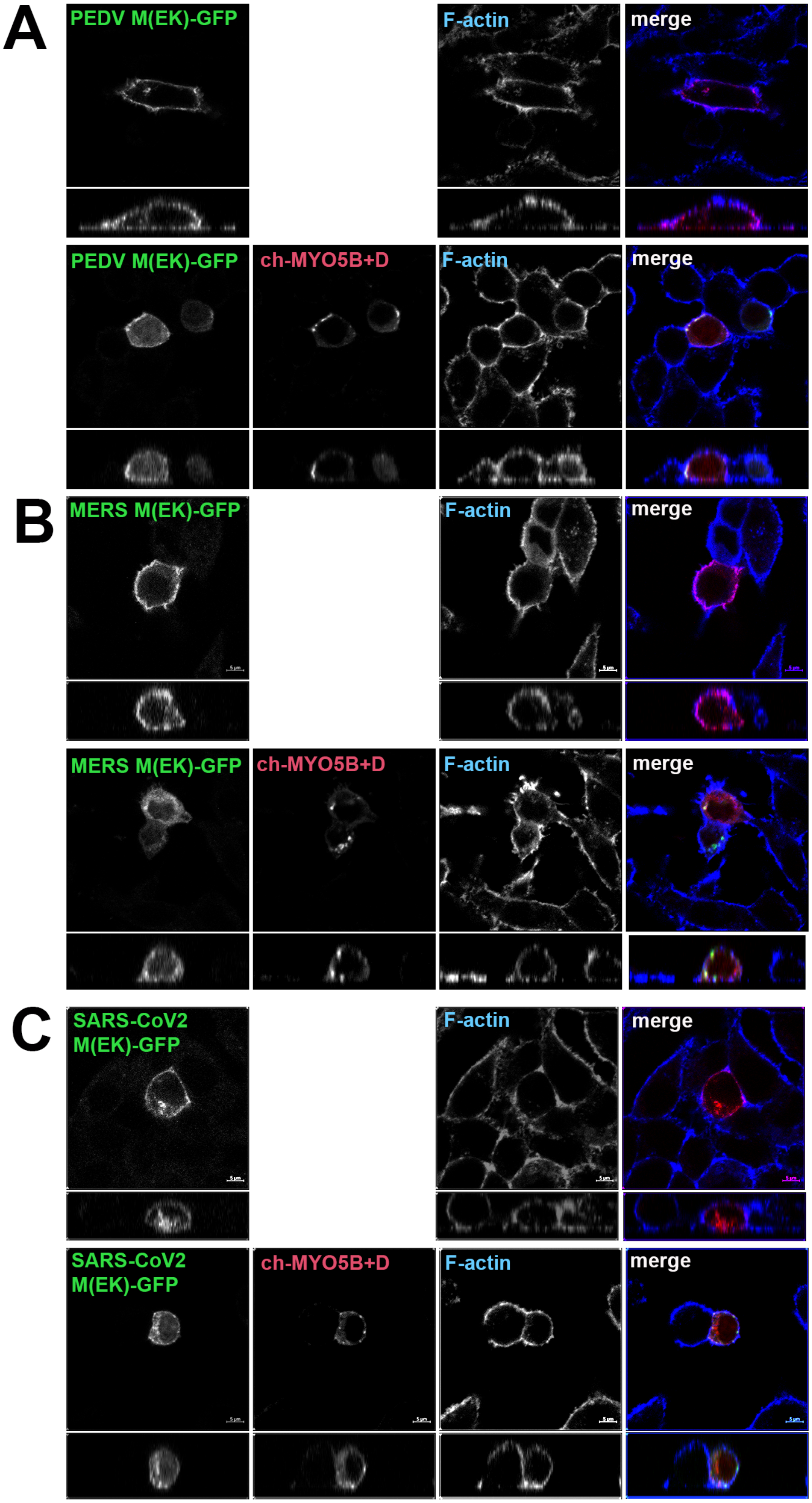
A
PEDV M(EK)-GFP
F-actin
merge
PEDV M(EK)-GFP
ch-MYO5B+D
F-actin
merge
B
MERS M(EK)-GFP
F-actin
merge
MERS M(EK)-GFP
ch-MYO5B+D
F-actin
merge
C
SARS-CoV2 M(EK)-GFP
F-actin
merge
SARS-CoV2 M(EK)-GFP
ch-MYO5B+D
F-actin
merge

Supplemental Figure S6: Loss of co-localization of coronavirus M proteins with co-expressed MYO5B+D with E to K mutations in A549 cells. E to K mutants of A) PEDV M-GFP, B) MERS M-GFP and C) SARS-CoV-2 M-GFP were expressed in A549 lung cells alone or with Cherry MYO5B+D. All cells were co-stained with phalloidin to visualize F-actin. Labels on the individual panels indicate the color used to produce the merged image. Z axis projections are shown below X-Y slice images, with the merged overlap at the right. Bar = 5 μm. Results are representative of 3 individual experiments.

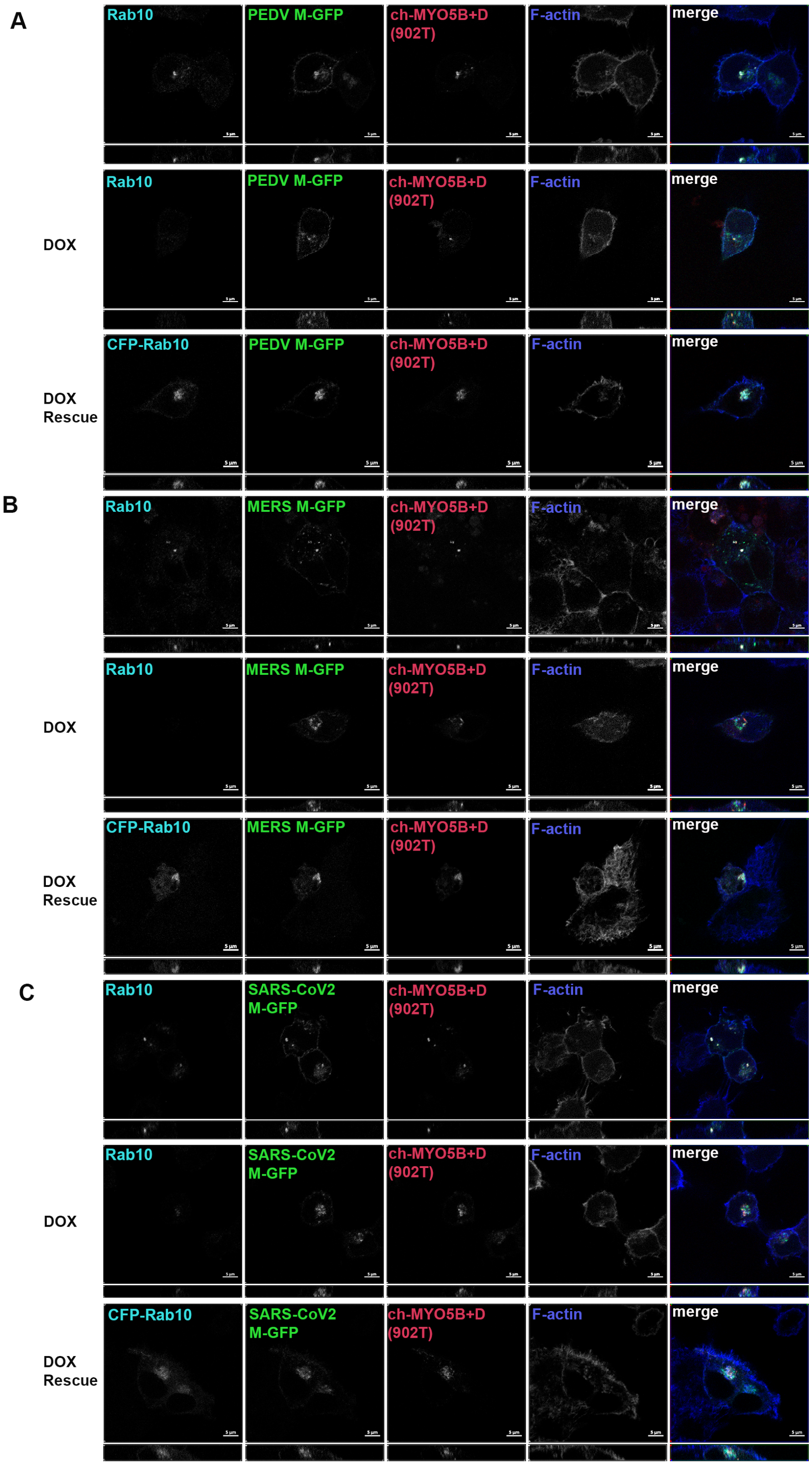
A
Rab10
PEDV M-GFP
ch-MYO5B+D (902T)
F-actin
merge
DOX
CFP-Rab10
DOX Rescue
B
MERS M-GFP
C
SARS-CoV2 M-GFP

Supplemental Figure S7: Knockdown of Rab10 expression in A549 cells alters localization of Coronavirus M proteins co-expressed with Cherry-MYO5B+D tail. A549 lung cells were transfected with A) PEDV M-GFP, B) MERS M-GFP and C) SARS-CoV-2 M-GFP with Cherry-MYO5B+D 902 tail (902T) in inducible Rab10 knockdown (KD) cells untreated or treated with doxycycline (DOX). One set treated Rab10 knockdown cells is rescued with the co-expression of CFP-Rab10. The untreated and treated (DOX) cells were co-immunostained for endogenous Rab10 and and phalloidin. The CFP-Rab10 rescued were only stained with phalloidin. Labels on the individual panels indicate the color used to produce the three-color merged image. Panels to the right of the merged panel were not used in the production of the merged image. Z axis projections are shown below X-Y slice images, with the merged overlap at the right. Bar = 5 µm. Results are representative of 3 individual experiments.
